# The effect of lung-conduction exercise in chronic obstructive pulmonary disease: Randomized, assessor-blind, multicenter pilot trial

**DOI:** 10.1097/MD.0000000000028629

**Published:** 2022-01-21

**Authors:** Su Won Lee, Jae Jun Park, Yee Ran Lyu, Eun Jung Lee, Si Yeon Kim, Weechang Kang, Ji Woong Son, In Chul Jung, Yang Chun Park

**Affiliations:** a Division of Respiratory Medicine, Department of Internal Medicine, College of Korean Medicine, Daejeon University, Daejeon, Republic of Korea; b Clinical Trial Center, Daejeon Korean Medicine Hospital of Daejeon University, Daejeon, Republic of Korea; c Korea Institute of Oriental Medicine, Republic of Korea; d Department of Rehabilitation Medicine of Korean Medicine, College of Korean Medicine, Daejeon University, Daejeon, Republic of Korea; e Department of Statistics, Hyehwa Liberal Arts College, Daejeon University, Daejeon, Republic of Korea; f Division of Respiratory and Critical Care Medicine, Department of Internal Medicine, Konyang University Hospital, Daejeon, Republic of Korea; g Department of Neuropsychology, College of Korean Medicine, Daejeon University, Daejeon, Republic of Korea.

**Keywords:** chronic obstructive pulmonary disease, Korean medicine, lung-conduction exercise, pulmonary rehabilitation

## Abstract

**Background:**

Pulmonary rehabilitation (PR) is a management modality that improves the quality of life of patients with chronic obstructive pulmonary disease (COPD); however, PR is not readily accessible. Therefore, we developed lung-conduction exercises (LCE) that can be performed easily without any limitations. The purpose of this randomized, assessor-blind, multicenter pilot trial was to compare the effects of LCE with PR and standard care (SC) in COPD patients.

**Methods:**

Twenty-five participants who met the eligibility criteria were randomly allocated to the SC group (only medication, n = 9), LCE group (medication + LCE, 5 times a week, n = 8), or PR group (medication + PR, 5 times a week, n = 8). The 6-minute walk distance (6WMD), pulmonary function test, modified Medical Research Council dyspnea scale, COPD assessment test (CAT), and St. George Respiratory Questionnaire (SGRQ) survey were carried out before starting the trial and after 4 and 8 weeks to determine motor performance, lung function, and dyspnea.

**Results:**

After 8 weeks, the pulmonary function test scores were the same. The 6MWD (PR, 28.3 ± 38.5; LCE, 14.5 ± 53.1; SC, 11.5 ± 20.5; *P* = .984), modified Medical Research Council dyspnea scale (PR, 0.8 ± 1.0; LCE, 0.8 ± 0.8; SC, 0.3 ± 0.5; *P* = .772), CAT (PR, 7.3 ± 6.2; LCE, 4.2 ± 5.2; SC, 1.0 ± 2.2; *P* = .232), and SGRQ scores (PR, 11.5 ± 15.4; LCE, 5.5 ± 13.1; SC, 4.8 ± 5.1; *P* = .358 [PR vs LCE], *P* = .795 [PR vs SC]) had improved in order of PR, LCE, and SC group. Although there were no statistically significant differences in the outcome measures between the groups, there were clinically significant improvements in the CAT and SGRQ scores.

**Conclusions:**

In this trial, PR showed more improvement in symptoms and quality of life than SC alone. To seek a more precise use of LCE, further full-sized studies with a long duration and additional outcome measures such as psychological assessment tools and cost-effectiveness ratio should be conducted.

**Trial registration:**

KCT0004724.

## 1 Introduction

Chronic obstructive pulmonary disease (COPD) exerts a heavy socioeconomic burden, with a prevalence rate of 12.16% reported in 2015,^[1]^ ranked fourth highest for mortality in 2000^[2]^ globally. COPD is defined as a common, preventable, and treatable disease characterized by persistent respiratory symptoms and airflow limitation that is due to airway and/or alveolar abnormalities, usually caused by significant exposure to noxious particles or gases.^[3]^ The airflow limitation is not fully reversible and the chronic inflammation of the airways and lung parenchyma are mainly triggered by smoking, occupational exposure, and infection.^[3]^ The symptoms of coughing, sputum, and dyspnea are often accompanied by negative emotions such as depression and helplessness, resulting in a decrease in quality of life because breathing has an absolute effect on daily life.^[3,4]^

Many pharmacological treatments have been developed for COPD patients.^[5]^ However, medications only target the symptoms and cannot prevent the progressive decline in lung function or manage other problems such as depression and muscle loss.^[6]^ The drugs used for anxiety and depression, common comorbidities in COPD, are known to have adverse effects (AEs) such as tremor, sweating, and confusion.^[7]^ Therefore, additional nonpharmacological treatments are required. Pulmonary rehabilitation (PR), a typical nonpharmacological treatment, is beneficial not only for improving symptoms but also for enhancing exercise capacity and treating depression and anxiety.^[8,9]^ However, PR also has limitations, as it needs to be provided by professionals and takes considerable time due to numerous hospital visits.^[10]^ Therefore, a self-controlling method that replaces PR is needed.

In previous studies, home-based PR, yoga, *tai chi*, and *qigong*, which can be considered alternatives that complement the application PR, have shown clinically significant improvements in symptoms and quality of life.^[11,12]^ However, these previous studies have limitations most did not have an intervention group with existing PR, and they had moderate–low quality of evidence due to the small number of trials.

Many ancient studies in Korean medicine have demonstrated methods and exercise therapies that can treat and prevent pulmonary diseases.^[13–15]^ Especially, “Dong-Ui-Bo-Gam”, an ancient medical literature approved by UNESCO as a cultural heritage in 2009, suggested *Taesikbub* and *Lung-doyinbub*.^[14]^*Taesikbeop* is a respiration method focused on taking deep breaths, and *Lung-doyinbeop* is a strengthening pulmonary exercise that includes the practicing of gymnastics, tapping, and breath-holding. We developed a lung-conduction exercise (LCE) that combines *Taesikbeop* and *Lung-doyinbeop* which can be performed by patients themselves in the comfort of their homes because they consist of simple movements.^[16]^ We hypothesized that LCE will be effective, via respiratory meditation, in emotionally stabilizing patients as well as relieving symptoms by increasing diaphragmatic elevation and force, ventilation efficiency, thoracic movements, and sputum discharge.^[17–22]^ This clinical trial was intended to determine the effects of LCE as a self-therapy and we anticipate that LCE is suitable for daily self-treatment, especially for older patients who have limitations with hospital visits. We used the 6-minute walk distance (6MWD), pulmonary function tests (PFTs), and several questionnaires as measured variables to evaluate exercise performance, symptoms, and quality of life. We planned a randomized, assessor-blind, multicenter trial to compare the effects of LCE with PR and of standard care (SC) in patients with moderate to severe COPD.

## 2 Methods

### 2.1 Study design and setting

This randomized, assessor-blind, parallel group, multicenter pilot trial was conducted at the Daejeon University Daejeon Korean Medicine Hospital and Konyang University Hospital in South Korea (CRIS.nih.go.kr, KCT0004724). This clinical trial consisted of LCE, PR, or SC. The enrolled participants who met the eligibility criteria were randomized to parallel groups at a ratio of 1:1:1 for the LCE, PR, and SC groups. The LCE or PR intervention was administered 5 times per week for 8 weeks. Assessments were performed at baseline (ie, 0-week) and after 4 and 8 weeks of intervention.

### 2.2 Participants

Patients with moderate and severe COPD, as diagnosed by forced expiratory volume in 1 s (FEV1)/forced vital capacity (FVC) <70% and FEV1 ≥30% but <80%, respectively, aged 40 to 80 years, were included in this trial. Participants who had complaints of difficulty in breathing at/above the modified Medical Research Council dyspnea scale (mMRC) ≥2 points and voluntarily agreed to participate in this clinical trial were included.

The exclusion criteria were as follows: patients with serious respiratory illnesses other than COPD (eg, lung cancer, pneumonia, active tuberculosis, tuberculosis pulmonary destruction, pneumonectomy, etc); unstable cardiovascular disease (unstable angina, acute myocardial infarction, severe aortic stenosis, etc), severe untreated pulmonary hypertension, history of acute deterioration within 2 weeks, change in FEV1 of 12% or forced vital capacity of 200 mL or more for 1 second before or after bronchodilator and asthma attack, illnesses that may cause death or disability in a 1-year period (eg, cancer, heart failure, coronary artery disease, cerebrovascular disease, kidney failure, diabetes with severe complications, uncontrolled hypertension, etc), and with difficulty walking (eg, due to cerebrovascular disease, osteoarthritis, and serious malnutrition); patients incapable of giving consent or unable to continue the study because of mental status change or other problems with intellect; pregnant or lactating women; alcoholics or those with a history of substance abuse; smokers; those who took medication in other clinical trials within 30 days before start of this trial (based on written consent); and those with an underlying disease deemed inappropriate for this trial by the investigators.

### 2.3 Randomization and blinding

An independent statistician used a random computer-generated number in SAS Analytics Pro 9.4 (SAS Institute, Cary, NC)^[23]^ for randomization. Subject identification codes (random numbers) were assigned to those who met the inclusion criteria and block randomization was performed. Participants were allocated to randomized and parallel groups at a ratio of 1:1:1 for the LCE, PR, and SC groups. The randomization table was maintained separately by the statistician until the trial was completed to maintain blinding and only the statistician had access to the random numbers by protecting the file from disclosure.

Because the participants and investigators cannot be blinded while performing the intervention, this was an assessor-blind trial. The assessor did not know what type of treatment the subject was receiving and evaluated the validity of the interventions.

Data collected at every visit in 2 hospitals was managed by case report form and collected finally at Daejeon University Daejeon Korean Medicine Hospital. Only the principal investigator or those who have permission was able to access the data. The copy of all clinical trial-related communications, the subjects’ records, consent, and case records has been kept in a controlled-access laboratory archive.

### 2.4 Intervention

#### 2.4.1 Lung-conduction exercise group

LCE is a Korean medicinal PR developed by Daejeon Korean Medicine Hospital of Daejeon University after reviewing the ancient Korean Medicine literature and consulting with experts.^[16]^ In the beginning, *Taesikbeop* was performed – taking a deep breath in and then partially breathing out, employing both diaphragmatic and pursed-lip breathing. *Taesikbeop* was performed 3 times to prevent airway obstruction and improve expiration by active and prolonged efficient breathing.^[17,18]^ By closing the eyes and focusing on breathing slowly, the respiratory rate per minute is reduced and blood circulation is improved, resulting in a relaxing effect.^[19]^ Subsequently, the patients exhaled whilst sitting on the ground with both hands and spine curled, and while inhaling, their chest would swell and they would rise back up. The movements of the chest and upper limbs increase the mobility of the thorax and spine and organized movements aid the upward and downward diaphragmatic breathing motions.^[20]^ The fist was then pounded on the left and right sides of the spine, similar to the percussion used for sputum discharge.^[21]^ The next steps were to hold the breath for a while and close the eyes, bump the teeth several times, and swallow as if saliva is stuck in the mouth – this was done to activate the brain and stimulate circulation to clears the mind and promote saliva secretion.^[22]^ Finally, after 3 more rounds of *Taesikbeop*, the mind is stabilized, and the exercise is completed. LCE took 20 minutes per day 5 times a week for a total of 8 weeks (Table 2 and Fig. 2).

### 2.5 Comparison

#### 2.5.1 Standard care group

Patients in this group received only standard medications. Medications were limited to long-acting muscarinic antagonists, long-acting beta-agonists or long-acting muscarinic antagonists and long-acting beta-agonists complexes, and short-acting beta-agonists, if necessary.

#### 2.5.2 Pulmonary rehabilitation group

Patients in this group received standard PR therapy based on the 2015 Respiratory Rehabilitation Guidelines published by the Korea Academy of Tuberculosis and Respiratory diseases.^[24]^ Patients performed warm-up, stretching, cardiovascular exercise (using an ergometer or treadmill walking), strength exercise, flexibility exercise, and cooling down. As for the main exercise, cardiovascular exercise using an ergometer or treadmill is effective in increasing walking distance, strengthening cardiopulmonary function, and increasing oxygen consumption in peripheral muscles.^[25]^ Strength exercise using dumbbells helps recondition skeletal muscles, and flexibility exercises including chest and upper and lower limb stretching improves chest mobilization and relaxation of postural tension.^[26]^ The intensity can be adjusted to the subject's ability. PR took 60 minutes per day, 5 times a week for 8 weeks (Table 1).

**Table 1:** Course of pulmonary rehabilitation.

No.	Course description	Time
1	Warming up consists of a low intensity (<40% maximal oxygen uptake) or medium intensity (40%–60% maximal oxygen uptake) activity	10 min
2	Stretching consists of whole-body relaxing activity	10 min
3	Main exercise consists of cardiovascular exercise (using an ergometer or treadmill), strength exercise of 60%–80% of the patient's maximum strength (ie, 1 set of 10–15 times of each muscle, set 2–3 times) and flexibility exercise (chest and upper and lower limbs stretching)	30 min
4	Warming down consists of cardiovascular and muscular endurance exercises with low intensity (<40% maximal oxygen uptake) or medium intensity (40%–60% maximal oxygen uptake)	10 min

**Table 2:** Course of lung-conduction exercise.

No.	Course description	Time
1	Sit up comfortably and slowly while inhaling through the nose. After sufficient inhalation, hold breath while counting as high as you can tolerate. Then gently breathe out through your mouth (set 3 times, gradually increasing the time of practice)	5 min
2	While sitting on the ground with both hands and spine curled, exhale and while inhaling, swell your back, and raise your back up (set 5 times)	4 min
3	Hold your fists, bend your arms behind your back and tap your spine to the left and right (set 15 times)	4 min
4	Hold your breath for a while, close your eyes, bump your teeth several times, and swallow if the saliva is stuck in your mouth (set 3 times)	2 min
5	Sit up comfortably and slowly while inhaling through your nose. After sufficient inhalation, hold breath, while counting as high as you can tolerate, and then gently breathe out with your mouth (set 3 times, gradually increasing the time of practice)	5 min

### 2.6 Ethics

This study was approved by the Institutional Review Board of the Daejeon University Daejeon Korean Medicine Hospital (DJDSKH-18-BM-19) and Konyang University Hospital (KYUH-2018-10-014-015). This clinical trial protocol followed all applicable regulations, including the ICH Good Clinical Practice Guidelines, the Helsinki Declaration (Seoul 2008), the Korean Good Clinical Practice Guidelines, the Korean Pharmaceutical Affairs Law, the Institutional Review Board, and data protection regulations.^[27]^

Written informed consent was obtained when the subjects decided to participate in the study. The investigators provided all the information relevant to the clinical trial, including the benefits and risks of participating in this study; the subjects signed a document containing all the instructions. The subject's identities were kept confidential at all times. All documents related to clinical trials, such as case records, were stored and distinguished by a subject identification code, not name. Only the monitors and inspectors involved in this clinical trial viewed the subject's records for the purpose of monitoring and managing the progress of the trial. All documents were kept confidential in a controlled-access laboratory archive.

### 2.7 Outcomes

#### 2.7.1 Primary outcome

The primary outcome in this study was the difference between the 3 groups in the 6MWD after week 4 and after completing the trial (ie, week 8) as compared to that baseline. The 6MWD test measures the total distance walked in 6 minutes. Patients were instructed to walk as much as possible for 6 minutes and they could rest or stop if needed. After the test, the total walking distance was calculated and recorded. The 6MWD test is an important measure of the exercise capacity of patients with COPD.^[28]^ The test was performed every 4 weeks (week 0, week 4, and week 8).

#### 2.7.2 Secondary outcomes

PFTs were performed before and after the trials (ie, at week 0 and week 8). mMRC score for respiratory distress, ranging from 0 to 4 points, is easy to use, and a highly reproducible indicator that can be used to select patients for rehabilitation.^[29]^ The COPD assessment test (CAT) is a short questionnaire for evaluating and monitoring COPD, with scores ranging from 0 to 40; it is sensitive to differences in state and provides a valid, reliable, and standardized measure of COPD health status.^[30]^ The mMRC and CAT scores were recorded at each visit point (week 0, week 4, week 8). The St. George Respiratory Questionnaire (SGRQ) was designed to measure health-related quality of life in patients with asthma and COPD, with scores from 0 to 100, and the validity and reliability of the Korean version of the SGRQ has been proven; the test was performed before and after the trials (ie, at week 0 and week 8). A score of 0 on the CAT and SGRQ represents the best quality of life and higher scores denote lower quality of life.

### 2.8 Safety

Safety assessment involved adverse effects (AEs), examination of vital signs, and clinical laboratory tests (liver function, routine blood, and urine tests). AEs and vital signs were recorded on a case report form at every visit, and clinical laboratory tests were conducted before and after the clinical trials. AEs were defined as symptoms not observed prior to trial intervention, including unintended symptoms, regardless of the trial. Investigators kept a complete record of symptoms, signs, duration, severity, relationship with the trial, measures, and outcomes of AEs.

### 2.9 Statistical analysis

Data analysis was performed by an independent statistician using in SAS Analytics Pro 9.4 (SAS Institute, Cary, NC).^[23]^ Continuous variables were summarized by mean ± standard deviation, and categorical variables were reported with frequencies and percentages. The effectiveness evaluation included the full analysis set based on the intention-to-treat principle, and per protocol (PP) analysis was the secondary analysis. The primary efficacy outcome measure, the 6MWD, was analyzed by repeated measures analysis of variance, and the secondary efficacy outcome measures, including PFT, mMRC, CAT, and SGRQ scores, were analyzed by analysis of variance using linear mixed models.

Safety evaluation was conducted with a group of subjects who received 1 or more interventions, and the assessor confirmed at least 1 safety-related data by visit or call after the trial intervention. A comparison of the number of AEs associated with the trial was performed using the Kruskal–Wallis test, and group comparisons of the proportion of subjects who experienced 1 or more AEs were carried out using the Pearson χ2 or Fisher exact test. Statistical significance, for primary outcome measure, secondary outcome measures, and safety evaluations, was set at the 5% significance level.^[23]^

## 3 Results

### 3.1 Participants

**Figure 1:**
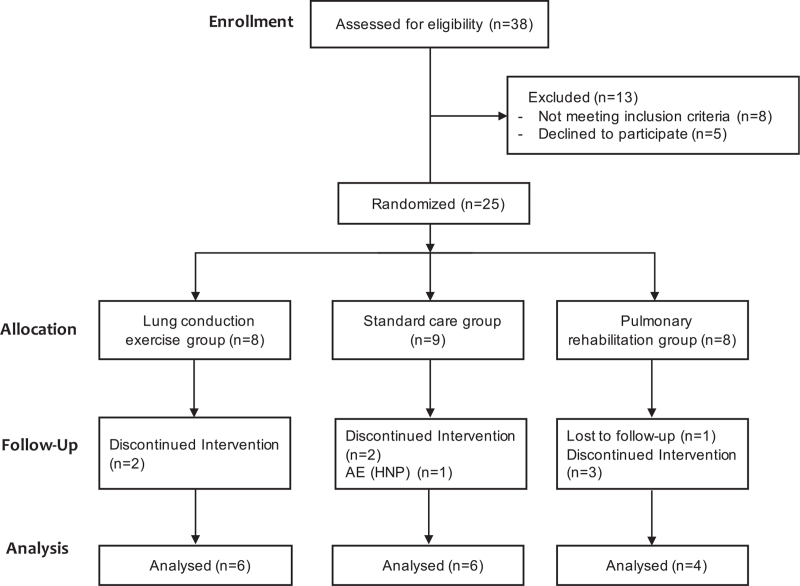
Flow chart of participants. AE = adverse event, HNP = herniated nucleus pulposus.

From January 2019 to August 2020, a total of 38 patients were screened and 25 participants were included in this study. Nine patients discontinued the study, so only 16 patients completed the study. Thus, there were 6 patients in the LCE group, 6 in the SC group, and 4 in the PR group (Fig. 1). There were no significant between-group differences in sex, age, weight, height, BMI, vital signs, and other outcome measures (Table 3).

**Figure 2:**
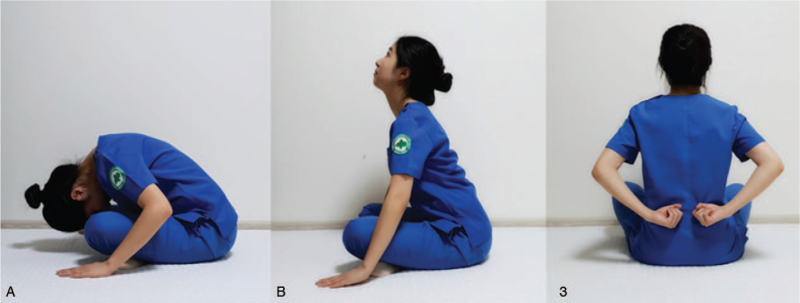
Course of lung-conduction exercise. 2-A: While sitting on the ground with both hands and spine curled, exhale. 2-B: While inhaling, swell your back, and raise your back up. 3: Hold your fists, bend your arms behind your back and tap your spine to the left and right.

**Table 3:** Baseline characteristics.

	LCE (n = 8)	SC (n = 9)	PR (n = 8)
Age (yr)	65.5 ± 9.6	70.8 ± 6.3	67.6 ± 10.9
Sex (N, male/female)	7/1	8/1	8/0
Weight (kg)	61.4 ± 15.4	73.1 ± 16.1	70.0 ± 10.1
Height (cm)	162.3 ± 6.1	167.3 ± 8.4	169.3 ± 5.5
BMI (kg/m^2^)	23.3 ± 5.4	25.0 ± 3.5	24.2 ± 3.1
Systolic blood pressure (mm Hg)	121.3 ± 16.0	128.9 ± 13.9	130.8 ± 24.7
Diastolic blood pressure (mm Hg)	74.1 ± 13.1	75.9 ± 11.6	80.4 ± 14.1
Pulse rate (N/min)	88.9 ± 14.0	82.8 ± 18.7	75.0 ± 12.4
Body temperature (°C)	36.7 ± 0.2	36.9 ± 0.1	36.9 ± 0.1
Ex-smoker (N)	6	7	5
FEV1 (L)	1.5 ± 0.5	1.6 ± 0.4	1.6 ± 0.4
FEV1/FVC (%)	50.4 ± 15.6	51.3 ± 10.5	47.3 ± 13.1
6MWD (m)	354.0 ± 58.7	375.8 ± 62.5	337.0 ± 79.6
mMRC	3.0 ± 0.9	2.3 ± 0.7	2.9 ± 1.0
CAT	24.5 ± 8.6	21.4 ± 9.1	20.4 ± 8.3
SGRQ	50.0 ± 17.8	41.4 ± 16.4	45.6 ± 15.0

### 3.2 Primary outcome

**Figure 3:**
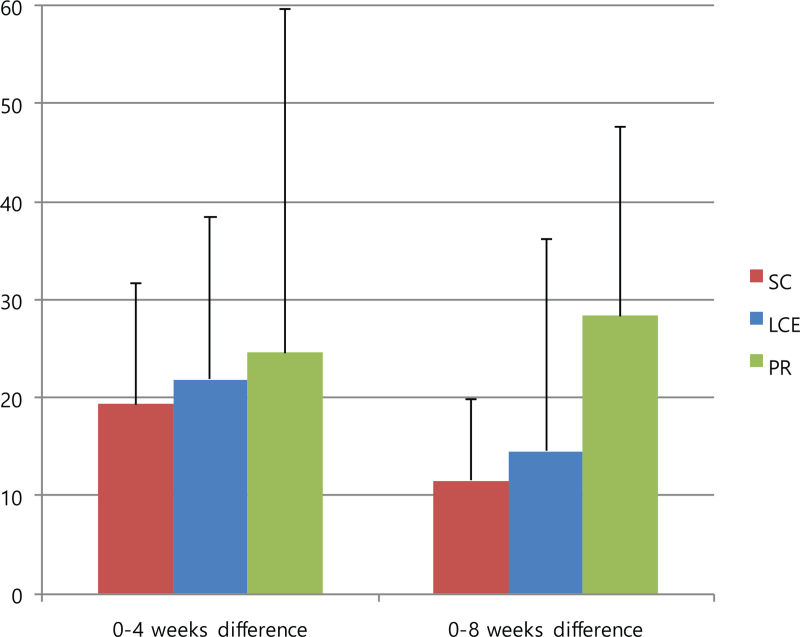
Mean differences in 6MWD from baseline to 4-weeks and from baseline to 8-weeks. Data are presented as mean values with error bars representing standard errors (95% CI, *P* = .984), FAS analysis. 6MWD = 6-minute walk distance, CI = confidence interval, FAS = full analysis set, LCE = lung-conduction exercise group, PR = pulmonary rehabilitation group, SC = standard care group.

The 6MWD was the primary outcome in this study, and the results are reported in Figure 3 and Table 4. The mean differences in the 6MWD from baseline to 4 weeks and from baseline to 8 weeks were not statistically significant between the groups (95% confidence interval, *P* = .984). However, all mean differences improved in the order of PR, LCE, and SC groups. For full analysis set, the mean difference in the 6MWD from baseline to 4 weeks increased by 24.6 ± 70.0 in the PR group, 21.8 ± 40.5 in the LCE group, and 19.3 ± 30.2 in the SC group, and from baseline to 8 weeks, it increased by 28.3 ± 38.5 in the PR group, 14.5 ± 53.1 in the LCE group, and 11.5 ± 20.5 in the SC group. For PP analysis, the mean difference from baseline to 4 weeks increased by 23.8 ± 80.8 in the PR group, 21.8 ± 40.6 in the LCE group, and 8.0 ± 23.8 in the SC group. On PP analysis, the mean 6MWD values from baseline to 4 weeks and 8 weeks were 337.0 ± 79.7 to 360.8 ± 64.7 and 365.2 ± 78.9, respectively, in the PR group, 354.0 ± 58.8 to 375.8 ± 62.5 and 368.5 ± 74.4, respectively, in the LCE group, and 387.0 ± 64.1 to 395.0 ± 57.9 and 398.5 ± 63.9, respectively, in the SC group.

**Table 4:** Mean differences in 6-minute walk distance (6MWD).

	0–4 weeks difference	0–8 weeks difference	*P*-value
LCE	21.8 ± 40.5	14.5 ± 53.1	.984
SC	19.3 ± 30.2	11.5 ± 20.5	
PR	24.6 ± 70.0	28.3 ± 38.5	

### 3.3 Secondary outcomes

**Figure 4:**
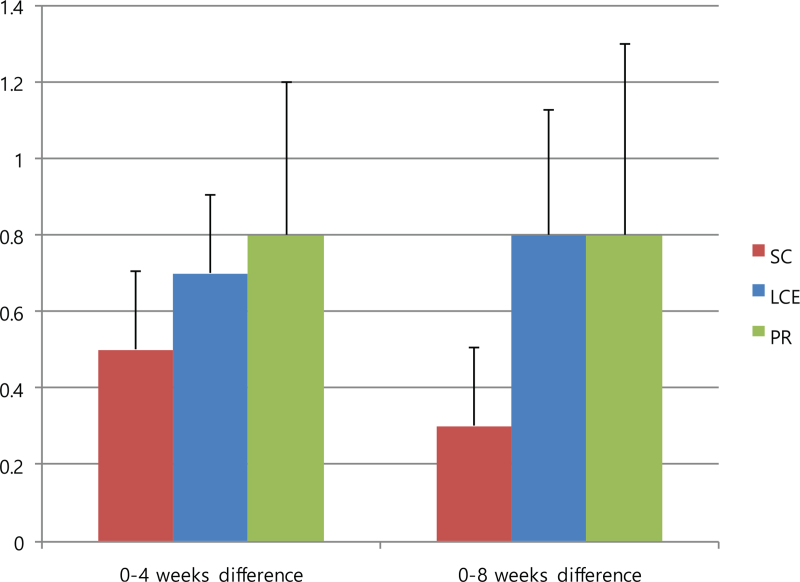
Mean differences in mMRC from baseline to 4-weeks and from baseline to 8-weeks. Data are presented as mean values with error bars representing standard errors (95% CI, *P* = .772). CI = confidence interval, LCE = lung-conduction exercise group, mMRC = modified Medical Research Council dyspnea scale, PR = pulmonary rehabilitation group, SC = standard care group.

The PFT results throughout the intervention period were sustained without statistically significant differences between groups (Table 5). The mean differences in the mMRC from baseline to 4 weeks and to 8 weeks were not statistically significant between the groups (95% confidence interval, *P* = .772). However, the mean difference in the mMRC from baseline to 4 weeks had improved in the following order: PR (0.8 ± 0.8), LCE (0.7 ± 0.5), SC (0.5 ± 0.5) group; from baseline to 8 weeks, the PR and LCE groups (0.8 ± 1and 0.8 ± 0.8, respectively) improved more than the SC group (0.3 ± 0.5) (Fig. 4).

**Table 5:** Results for pulmonary function test (PFT).

	0 weeks	8 weeks	Mean difference	*P*-value
FEV1 (L)				
LCE (n = 6)	1.6 ± 0.5	1.6 ± 0.6	−0.0 ± 0.1	.680
SC (n = 6)	1.7 ± 0.5	1.8 ± 0.6	0.1 ± 0.2	.626
PR (n = 4)	1.7 ± 0.5	1.7 ± 0.7	0.0 ± 0.4	
FEV/FVC (%)				
LCE (n = 6)	53.8 ± 16.5	54.5 ± 18.5	0.7 ± 2.9	.376
SC (n = 6)	55.3 ± 10.6	55.0 ± 10.9	−0.3 ± 4.1	.209
PR (n = 4)	47.3 ± 17.7	49.8 ± 21.7	2.5 ± 6.2	

**Figure 5:**
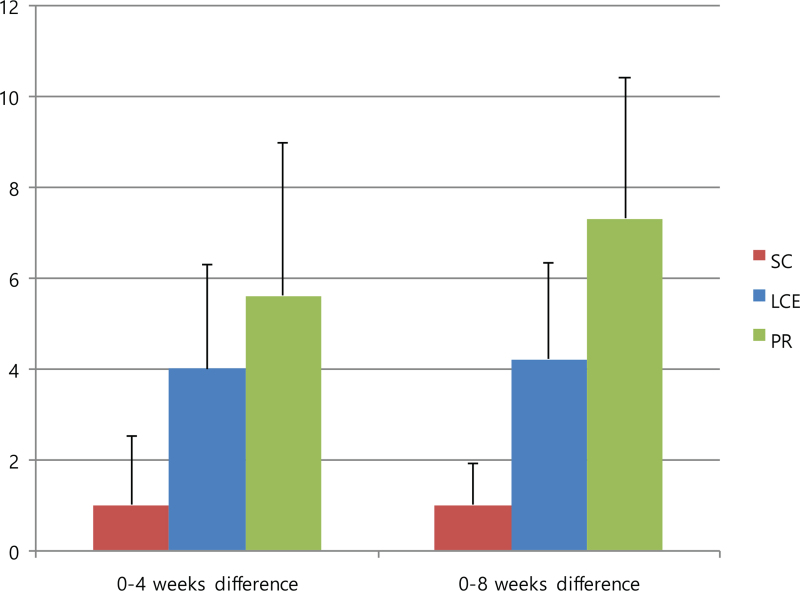
Mean differences in CAT from baseline to 4-weeks and from baseline to 8-weeks. Data are presented as mean values with error bars representing standard errors (95% CI, *P* = .232). CI = confidence interval, CAT = chronic obstructive pulmonary disease assessment test, LCE = lung-conduction exercise group, PR = pulmonary rehabilitation group, SC = standard care group.

**Figure 6:**
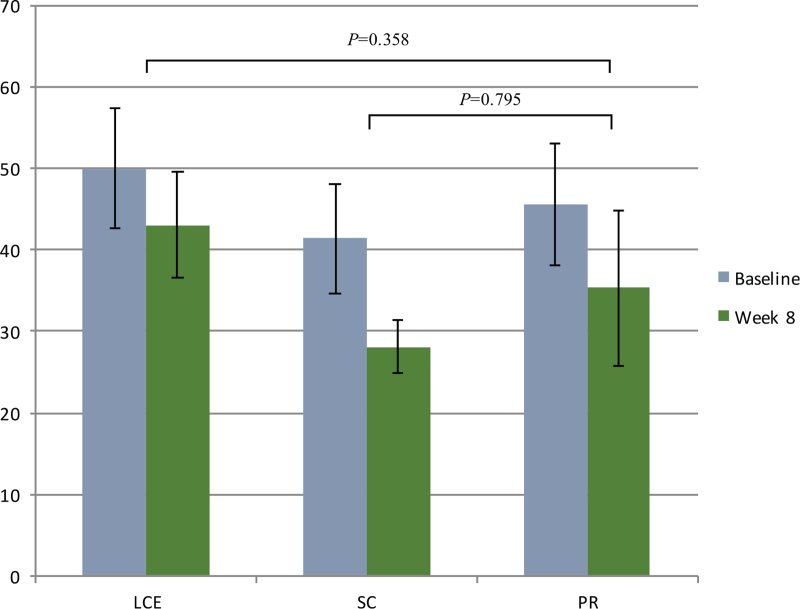
Results for SGRQ from baseline to 8-weeks. Data are presented as mean values with error bars representing standard errors (95% CI). CI = confidence interval, LCE = lung-conduction exercise group, PR = pulmonary rehabilitation group, SC = standard care group, SGRO = St. George Respiratory Questionnaire.

Although there were no statistically significant differences in the CAT and SGRQ scores from baseline to 4 weeks and 8 weeks between the groups, there were clinically significant improvements in the PR and LCE groups. The minimum clinically important differences (MCIDs) for CAT and SGRQ were 2 and 4, respectively.^[31,32]^ The mean differences from baseline to 4 weeks in the CAT scores were 5.6 ± 6.7 in the PR group, 4.0 ± 5.6 in the LCE group, and 1.0 ± 3.7 in the SC group and from baseline to 8 weeks, it was 7.3 ± 6.2 in the PR group, 4.2 ± 5.2 in the LCE group, and 1.0 ± 2.2 in the SC group (Fig. 5). Moreover, the mean difference in the SGRQ from baseline to 8 weeks was 11.5 ± 15.4 in the PR group, 5.5 ± 13.1 in the LCE group, and 4.8 ± 5.1 in the SC group. The mean difference in SGRQ scores from baseline to 8 weeks was 45.6 ± 15.0 to 35.3 ± 19.1 in the PR group, 50.0 ± 17.8 to 43 ± 15.9 in the LCE group and 41.4 ± 16.4 to 28.1 ± 7.8 in the SC group (Fig. 6).

### 3.4 Adverse events

No AEs related to this study were reported; 2 unrelated AEs were reported: toe line fracture caused by patient carelessness elsewhere and herniated nucleus pulposus due to exacerbation of the underlying disease. In addition, patients with herniated nucleus pulposus were in the control group, and there was no causal relationship with this trial. The vital signs and clinical laboratory test results showed no significant changes before and after the clinical trial.

## 4 Discussion

The trends of increasing prevalence of and mortality from COPD are expected to continue because of the increasing aging population and the preponderance of risk factors.^[33]^ The need for daily management of problems such as depression, muscle weakness, and weight loss that medications cannot handle is also increasing. PR is suitable for daily management^[34]^ and clinical trials suggest that PR relieves dyspnea and fatigue, improves emotional function, and enhances the sense of control that individuals have over their condition.^[35]^ However, its execution rate is low due to lack of recognition, professionals, facilities, and so on.^[24]^ Therefore, we developed the LCE,^[16]^ a PR method based on Korean medicine that consists of simple movements and is available regardless of facilities. The aim of this pilot study was to evaluate the efficacy and safety of LCE compared with that PR and SC.

In previous studies of home-based PR similar to LCE, home-based PR was found to improve the quality of life and exercise capacity of COPD patients as well as relieve dyspnea status and reduce hospital readmission rates.^[11]^ Home-based PR tended to have a lower treatment effect than hospital-based PR in several studies,^[8]^ in most studies, there was only a control group and no intervention group with hospital-based PR.^[11]^ Nevertheless, home-based PR is an encouraging alternative to hospital-based PR and has benefits, especially for those with severe COPD who are housebound or who cannot afford or do not prefer hospitalization. Moreover, home-based PR has higher compliance than hospital-based PR because several factors such as the inconvenience and cost of traveling to hospital in hospital-based PR directly affect patient compliance, and low-intensity home-based PR is easy for patients to maintain.^[36]^ LCE is also considered to have similar advantages in that it is positive for elderly housebound patients and has high compliance.

Similarly, there are active mind–body movement therapies (AMBMTs) such as yoga, *tai chi*, and *qigong*, which are considered alternatives to PR. In a meta-analysis of studies comparing AMBMTs and PR alone, AMBMTs showed statistically significant improvements in SGRQ and CAT scores; further, AMBMT plus PR led to significant improvements in generic quality of life than PR alone.^[12]^ However, walking training was the only component of PR in most studies and there was a small number of randomized controlled trials on PR, which lowered the quality of evidence in this meta-analysis.^[12]^

Based on the Korean Respiratory Rehabilitation Guidelines, the existing PR consisted of breathing training, namely pursed-lip breathing and diaphragmatic breathing, cardiovascular exercise, strength exercise, and flexibility exercise. LCE also has the advantages of PR as it is composed of breathing training that includes pursed-lip breathing and diaphragmatic breathing, chest mobilizing exercise, and sputum discharge training. In addition, it has the features of breathing meditation and mind–body training; we expect it to contribute to psychological stability and symptom relief.

Although the results of this clinical trial should be interpreted with caution as it is a pilot study, it should be noted that the outcome measures improved in order of PR, LCE, and SC groups. The primary results of this study suggest that LCE has a positive effect on patients with COPD than SC alone. As for the primary outcome measure, 6MWD, PP analysis showed that the increase in mean difference from baseline to 4 weeks was similar in the PR group (23.8 ± 80.8) and LCE group (21.8 ± 40.6), but it was less than that in the SC group (8.0 ± 23.8). The mean difference from baseline to 8 weeks was higher in the PR group (28.3 ± 38.5) than the SC group (11.5 ± 20.5) and LCE group (14.5 ± 53.1). In the LCE group, the 6WMD of 8 weeks was smaller than 4 weeks because of 1 or 2 patients’ influence within the small sample size. For the second outcome measure, the PFT results did not show significant changes among PR, LCE, and SC groups as observed in previous studies.^[37]^ The mean difference in the mMRC score from baseline to 4 weeks and to 8 weeks had also improved in the order of PR, LCE, and SC groups. In particular, there were clinically significant improvements in the CAT and SGRQ scores of the PR and LCE groups, indicating their effectiveness in improving symptoms and quality of life. The proper clinical interpretation in a trial should consider not only statistical significance but also whether the observed change is meaningful to patients.^[38]^ The MCID is a patient-centered concept, seizing both the proportion of the improvement and also the value patients place on the change.^[38]^ The mean difference in CAT scores from baseline to 8 weeks was 7.3 ± 6.2 in the PR group and 4.2 ± 5.2 in the LCE group, with scores exceeding the MCID of 2. Moreover, the mean difference in SGRQ scores from baseline to 8 weeks was 11.5 ± 15.4 in the PR group and 5.5 ± 13.1 in the LCE group, with scores exceeding the MCID of 4. In addition, 4 out of 8 patients in the PR group discontinued intervention or were lost to follow-up, whereas 2 out of 8 patients in the LCE group discontinued the intervention, showing higher compliance in the LCE group than the PR group. This difference in compliance reflects the convenience of intervention which is an important point for severe COPD patients to persist in exercise.

This study has several limitations. The first is the small sample size and rather short study duration. As it is the pilot study to examine the feasibility of various aspects of the study for a larger, confirmatory investigation, we recruited comparatively small samples to achieve the purpose of this pilot trial. We plan to calculate the sample size according to this study results to determine the effect size. The sample size required for a full-sized RCT can be assumed according to the results of the 6MWD. In order to determine the effect size, at least 20 to 30 participants per group are required to elicit a significant change.^[36,39]^ As for the duration, the outcome measures in this pilot study improved from 4 weeks to 8 weeks, and most programs are 12 week long; thus, a duration of 12 weeks would be better to assess the effect.^[8]^ To evaluate the maintenance duration (how long the treatment effects persists) of LCE and PR and to compare the long-term compliance to LCE and PR, it would be necessary to follow the patients for about 6 months.

Second, there is a lack of outcome measures that can specifically assess the effects of LCE. We hypothesized that LCE would be effective, via respiratory meditation, in emotionally stabilizing patients. However, we did not use psychological assessment tools such as the depression rating scale. In addition, like the home-based PR mentioned earlier, LCE can also save transportation costs and travel time for hospital visits. The cost-effectiveness ratio of LCE vs PR needs to be determined. Future studies can provide significant results by increasing the number of subjects, extending the study duration, and adding appropriate outcome measures. In addition, future studies can identify effective components of the program, the ideal program composition, and appropriate degree of supervision.

Third, although LCE has the advantage of availability and simple movements, it has the disadvantage insufficient amount of exercise compared to PR. While PR takes 60 minutes and consists of warm-up, stretching, cardiovascular exercise, strength exercise, flexibility exercise, and warm-down, LCE takes 20 minutes and is composed of relatively easy movements such as breathing training, chest mobilizing exercise, sputum discharge training, and meditation. Thus, LCE is suitable for daily self-treatment, especially for elderly housebound patients and not for patients with moderate disease severity who can visit the outpatient hospital frequently and are willing to pay for treatment. Therefore, as an additional method for the latter, it would be beneficial to combine the strengths of PR and LCE by adding cardiovascular exercise and strength exercise to LCE. Additionally, Chuna manual therapy that relaxes the breathing muscles can be added to combination of PR and LCE under the Korean traditional PR program. In practical, there is a case study of retrospective observation by applying this program clinically.^[40]^

Despite the limitations mentioned above, this study is the first clinical trial to demonstrate the effect of LCE against standard PR and SC. This pilot trial investigated the feasibility of the LCE intervention for COPD patients and provided clinical evidence before a further large-scale trial. The results of this study suggest that LCE is more beneficial for patients with COPD than SC. Although there were no statistically significant differences in outcome measures between groups, there were clinically significant improvements in the PR and LCE groups in terms of symptoms and quality of life. The fact that the change for the CAT and SGRQ scores in the LCE group was higher than the MCIDs suggests that the LCE is beneficial to COPD patients and is worth applying. Besides, high compliance in the LCE group indicates that COPD patients can continue the treatments and lengthen maintenance duration. LCE can be used for elderly housebound patients who cannot afford hospital-based PR to improve symptoms and quality of life, and we also recommended developing new programs by adding other therapies from Korean medicine for patients with moderate disease severity.

## 5 Conclusion

PR and LCE showed clinically significant improvements in all outcome measures indicative of symptoms and quality of life as opposed to SC alone. Especially, the CAT and SGRQ scores changes in the LCE group were higher than MCIDs meaningful to patients. The limitation of this study is that there were no statistically significant differences in the outcome measures between the groups, and the number of included subjects was not large to yield significant differences since is the pilot study. Therefore, when we conduct next clinical trial with large samples, we will add outcome measures with a longer duration to improve the limitations of this pilot study.

## Author contributions

**Conceptualization:** Ji Woong Son, In Chul Jung.

**Data curation:** Su won Lee, Eun Jung Lee, Si Yeon Kim, Weechang Kang, Ji Woong Son, In Chul Jung.

**Formal analysis:** Si Yeon Kim, Weechang Kang.

**Investigation:** Su won Lee, Jae Jun Park, Yee Ran Lyu, Eun Jung Lee, Si Yeon Kim, Weechang Kang.

**Project administration:** Yang Chun Park.

**Software:** Si Yeon Kim, Weechang Kang.

**Supervision:** Ji Woong Son, In Chul Jung, Yang Chun Park.

**Writing – original draft:** Su won Lee, Jae Jun Park.

**Writing – review & editing:** Yee Ran Lyu, Yang Chun Park.
